# Phylogenetically Distinct Near-Complete Genome Sequences of Porcine Reproductive and Respiratory Syndrome Virus Type 2 Variants from Four Distinct Disease Outbreaks at U.S. Swine Farms over the Past 6 Years

**DOI:** 10.1128/MRA.00260-21

**Published:** 2021-08-19

**Authors:** Declan C. Schroeder, Nkechi M. Odogwu, Jessica Kevill, My Yang, Venkatramana D. Krishna, Mariana Kikuti, Nakarin Pamornchainavakul, Carles Vilalta, Juan Sanhueza, Cesar A. Corzo, Albert Rovira, Scott Dee, Eric Nelson, Aaron Singrey, Perle Zhitnitskiy, Cecilia Balestreri, Dennis N. Makau, Igor A. D. Paploski, Maxim C.-J. Cheeran, Kimberly VanderWaal, Montserrat Torremorell

**Affiliations:** a Department of Veterinary Population Medicine, University of Minnesota, St. Paul, Minnesota, USA; b School of Biological Sciences, University of Reading, Reading, United Kingdom; c Veterinary Diagnostic Laboratory, University of Minnesota, St. Paul, Minnesota, USA; d Pipestone Applied Research, Pipestone, Minnesota, USA; e Animal Disease Research and Diagnostic Laboratory, South Dakota State University, Brookings, South Dakota, USA; f Department of Veterinary and Biomedical Sciences, South Dakota State University, Brookings, South Dakota, USA; Portland State University

## Abstract

Porcine reproductive and respiratory syndrome virus (PRRSV) continues to mutate, causing disruptive PRRS outbreaks in farms that lead to reproductive failure and respiratory disease-associated mortality. We present four new PRRSV type 2 variants in the United States belonging to four distinct *orf5* sublineages within lineage 1.

## ANNOUNCEMENT

**FIG 1:**
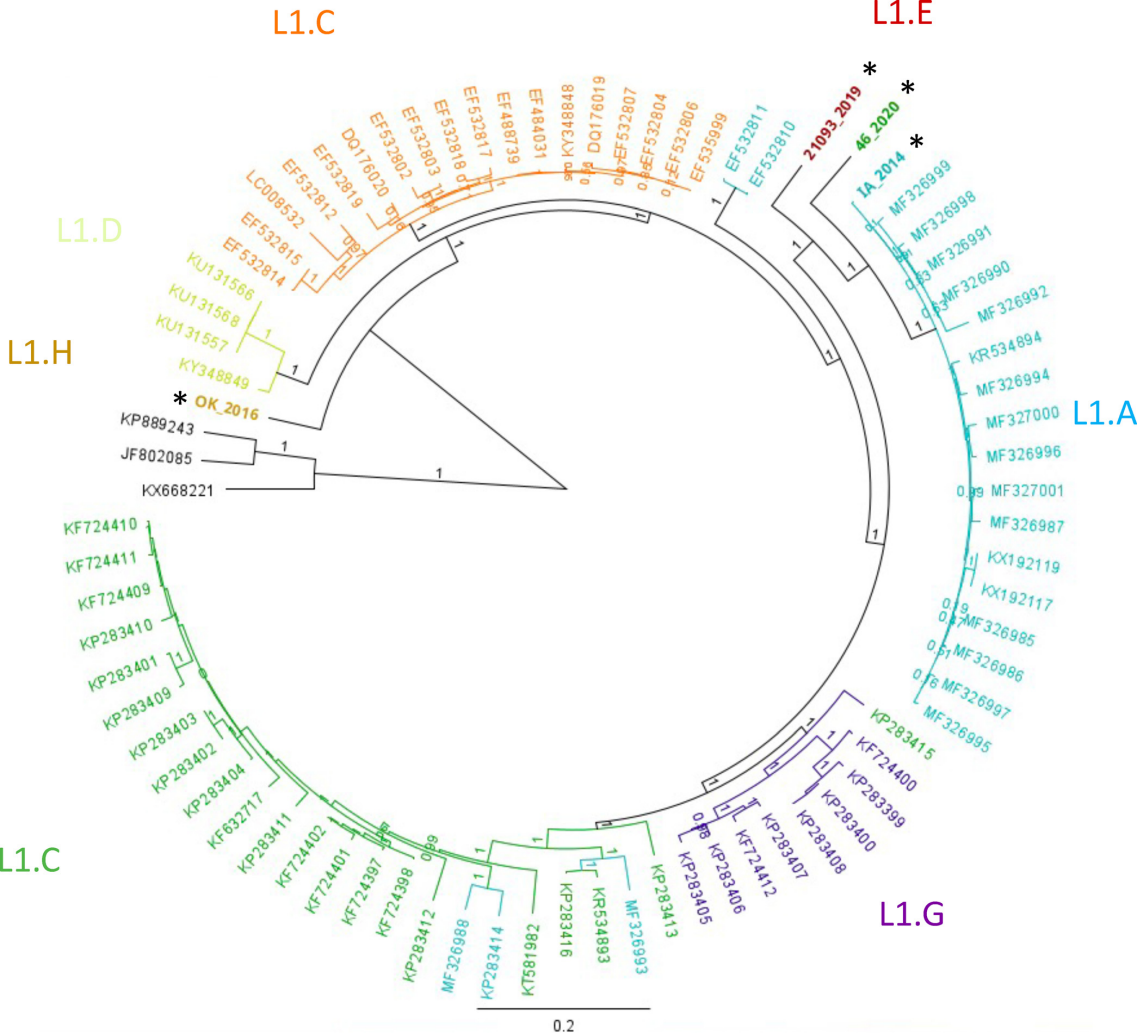
Phylogenetic inference tree showing new PRRSV genome sequences (*) generated from this study related to PRRSV lineage 1 complete genome sequences from ORF1 to ORF7. The genomes were aligned using Muscle 3.8.425 and visualized using FastTree 2.1.11 in Geneious Prime 2021.1.1. The tree was rooted with PRRSV-1 species in black, and the L1 sublineages were colored to match known L1 *orf5* sublineages. All *orf5* L1 sublineages were assigned based on an *orf5* phylogeny using the assignments by Paploski et al. (6). The bar represents 2 mutations per 10 nucleotides.

Porcine reproductive and respiratory syndrome virus (PRRSV) is a positive single-stranded RNA virus and a member of the family *Arteriviridae* (1). The first cases of PRRS occurred almost simultaneously in both the midwestern United States and Western Europe in the late 1980s (2–4), with PRRSV being identified as the etiological agent of PRRS (4, 5). New PRRSV variants emerge and reemerge routinely throughout the United States and Europe (6, 7). Here, we report the genome sequences from four PRRSV variants associated with farm-level outbreaks spanning 6 years. Phylogenetic classification using the open reading frame 5 (*orf5*) gene places these variants in lineage 1 (L1; Fig. 1) (6, 8). Two PRRSV variants were isolated using the method described by Collins et al. (5), except that MARC-145 cells were used for the isolation, propagation, and enumeration (9). These two variants came from outbreaks in the Midwest and south-central United States in 2014 (10) and 2016, designated IA/2014 and OK/2016, respectively. While the IA/2014 genome sequence was assembled from cDNA synthesized from RNA extracted from a MARC-145 infected cell culture (1.31 × 10^6^ 50% tissue culture infective doses [TCID_50_]/ml), the OK/2016 assembly came from cDNA synthesized from a lung tissue RNA sample that was harvested from an intramuscularly inoculated naive pig (5 × 10^3^ TCID_50_/ml) with a cycle threshold (Ct) value of 27 (11) (serum sample taken at the time of necropsy) that was humanely euthanized at day 14 postinoculation. The third and fourth genomes, 21093/2019 and 46/2020, were assembled from cDNA synthesized from RNA extracted from field serum and lung tissue samples, respectively. The lung tissue sample was collected from a pig that was submitted for necropsy at the University of Minnesota Veterinary Diagnostic Laboratory.

RNA was extracted per the manufacturer’s instructions using the MagMAX-96 viral RNA isolation kit (Applied Biosystems, Thermo Fisher Scientific) from isolates propagated in cell culture (IA/2014) or tissue (OK/2016) and field-collected serum (21093/2019). The NucleoSpin RNA virus kit (TaKaRa Bio USA, Inc.) was used on tissue collected from a necropsied animal (46/2020). Four independently created cDNA libraries were generated using the SMARTer universal low-input RNA kit for first-strand synthesis using a custom-designed universal PRRSV-specific primer (5′-CCCTAATTGAATAGGTGACTTAG-3′) and the PrimeSTAR GXL polymerase for second-strand synthesis (TaKaRa Bio USA, Inc.). The Oxford Nanopore Technologies (ONT) libraries were prepared using the ligation (SQK-LSK109 for 21093/2019), rapid (SQK-RAD004 for OK/2016), or rapid barcoding (SQK-RBK004 for IA/2014 and 46/2020) sequencing kits. All libraries were individually barcoded, where appropriate, and sequenced using the high-accuracy base-calling model with a minimum Q score of 7 set on an ONT MinION or GridION sequencing platform using multiple FLO-MIN106 R9 flow cells. The genome assembly and phylogenies were created using default parameters for all software. The reads obtained were reference assembled against a small set of PRRSV lineage 1 genome sequences (GenBank accession numbers MN175677, KX758249, MF326988, and KT581982) using Minimap 2.17 in Geneious Prime 2021.1.1 and manually curated to correct ambiguities where possible. For each genome (IA/2014, OK/2016, 21093/2019, and 46/2020), a total of 157,384, 13,098, 41,223, and 27,378 reads were used to assemble genome sequences of 15,059, 14,952, 14,977, and 15,122 kb, respectively. The ORF5 gene from each genome was used to assign the L1 sublineages. The sequences of ORF1 to ORF7 from the new genomes were aligned to available lineage L1 PRRSV genomes using Muscle 3.8.425 and visualized using FastTree 2.1.11 in Geneious Prime 2021.1.1 (Fig. 1).

The IA/2014 genome clusters with other L1.A *orf5* sublineages (Fig. 1). The variants OK/2016 and 21093/2019 were assigned to the newly described L1.H and L1.E *orf5* sublineages (6), respectively. However, both are orphans in the ORF1 to ORF7 tree (Fig. 1), as no similar complete genome sequence for these two sublineages had been deposited to GenBank as of June 2021. Finally, the 2020 genome, 46/2020, classified as the L1.C *orf5* sublineage, did not cluster with other L1.C *orf5* sublineage genotypes (Fig. 1). To our surprise, the phylogeny of complete coding genomes does not correspond to the *orf5* phylogeny, as sequences assigned to the L1.C and L1.A sublineages are polyphyletic (Fig. 1).

### Data availability.

The genome sequences for this project have been deposited in GenBank under the following accession numbers: MZ423533 (IA/2014), MZ423534 (OK/2016), MZ423536 (21093/2019), and MZ423535 (46/2020). The Oxford Nanopore Technology reads are available under BioProject accession number PRJNA738550.
